# Accelerating Neutron Tomography experiments through Artificial Neural Network based reconstruction

**DOI:** 10.1038/s41598-019-38903-1

**Published:** 2019-02-21

**Authors:** Davide Micieli, Triestino Minniti, Llion Marc Evans, Giuseppe Gorini

**Affiliations:** 1 Università della Calabria, Dipartimento di Fisica, Arcavacata di Rende (Cosenza), 87036 Italy; 2 Università degli Studi Milano-Bicocca, Dipartimento di Fisica “G. Occhialini”, Milano, 20126 Italy; 3 STFC, Rutherford Appleton Laboratory, ISIS Facility, Harwell, United Kingdom; 4 Culham Centre for Fusion Energy, Culham Science Centre, Abingdon, Oxfordshire United Kingdom; 5 College of Engineering, Swansea University, Bay Campus, Fabian Way, Swansea, United Kingdom

## Abstract

Neutron Tomography (NT) is a non-destructive technique to investigate the inner structure of a wide range of objects and, in some cases, provides valuable results in comparison to the more common X-ray imaging techniques. However, NT is time consuming and scanning a set of similar objects during a beamtime leads to data redundancy and long acquisition times. Nowadays NT is unfeasible for quality checking study of large quantities of similar objects. One way to decrease the total scan time is to reduce the number of projections. Analytical reconstruction methods are very fast but under this condition generate streaking artifacts in the reconstructed images. Iterative algorithms generally provide better reconstruction for limited data problems, but at the expense of longer reconstruction time. In this study, we propose the recently introduced Neural Network Filtered Back-Projection (NN-FBP) method to optimize the time usage in NT experiments. Simulated and real neutron data were used to assess the performance of the NN-FBP method as a function of the number of projections. For the first time a machine learning based algorithm is applied and tested for NT image reconstruction problem. We demonstrate that the NN-FBP method can reliably reduce acquisition and reconstruction times and it outperforms conventional reconstruction methods used in NT, providing high image quality for limited datasets.

## Introduction

Neutron Tomography (NT) is a well established technique that provides the map of the neutron attenuation coefficients within an object^1,2^. Neutron Imaging (NI) is especially well suited to study for example thick metals, hydrogenous materials and porous media^3^, hence found applications in biology^4–6^, agriculture^7,8^, archaeology^9,10^, material science and engineering^11^. In some cases NI offers incomparable results with respect to X-ray imaging. Computed Tomography (CT) consists in acquiring many transmission images of the sample at different angle views. Subsequently, the three-dimensional map of the attenuation coefficients is computed starting from the projection data by means of a mathematical reconstruction algorithm. Long scan times - generally several hours, depending on the sample and the desired spatial resolution - are required to perform neutron measurements, since the flux of the existing neutron sources is several order of magnitude lower than the X-ray sources. NT is a useful tool for evaluating the structural integrity of objects but it is time-consuming and therefore not feasible to scan large quantities of similar samples, such as in quality check systems. Hence, in the NT field there is great interest in the reduction of scan time, motivated also by the high neutrons production cost.

One way to reduce the CT scan time is to limit the number of projections. Analytical reconstruction algorithms, e. g. the widely used Filtered Back-Projection (FBP) algorithm, generate streaking artifacts in the reconstructed images when the number of projections does not satisfy the Nyquist-Shannon condition^12,13^. Conversely, iterative algorithms better handle limited and noisy datasets, providing higher reconstruction quality than analytical algorithms^14^. In fact, they rely on a discrete representation of the image formation process, described by a linear system of equations solvable through an iterative optimization method. A wide variety of regularized iterative methods have been proposed in literature^15–19^. This class of reconstruction algorithms involve additional regularizing terms in the objective function. The prior knowledge about the scanned sample is embedded in the regularizing term, providing accurate reconstruction from high under-sampled datasets. Recently, the performances of the FBP algorithm and different iterative reconstruction methods were tested with neutron data and discussed^20^. Although iterative methods generally outperform analytical ones to handle limited-data problems, they present two major drawbacks. The first is the high computational cost, several order of magnitude greater than analytical methods. The latter is the limited variety of samples that can be reconstructed, due the constrain imposed by the specific prior knowledge. For example, Total Variation minimization based methods can be used only to accurately reconstruct objects with sparse gradient. Hence, the application to large-scale tomographic data is still limited.

Nowadays, Deep Learning^21^ (DL) has reached state-of-the-art performance for image classification^22–24^, segmentation^25–27^, image denoising^28–30^, deconvolution^31^ and artifact reduction^32,33^. Recently, new Machine Learning (ML) based methods were introduced to improve low-dose and Sparse-View X-ray tomography^34–41^. These methods are data-driven, i.e. they learn the image features from training data providing more accurate reconstructions than analytical methods.

**Figure 1:**
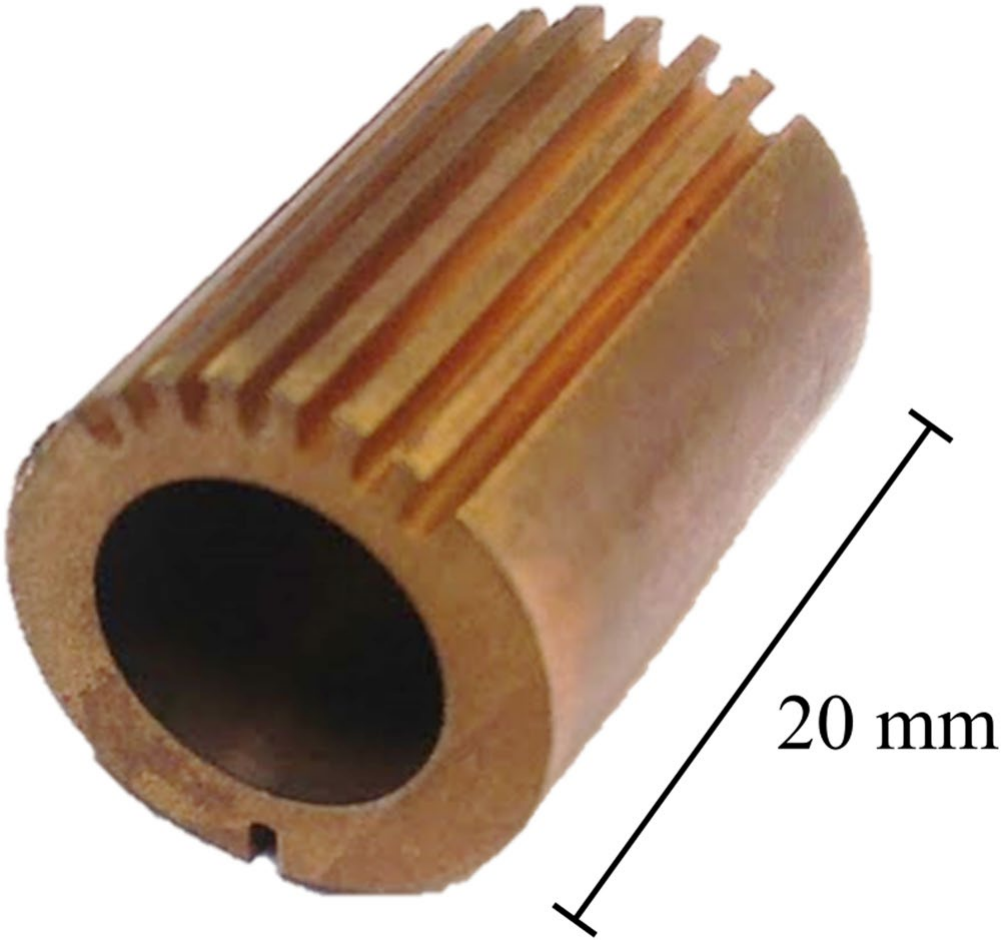
The sample inspected using NT. The Cu-CuCrZr pipe is the central section of the Culham Centre for Fusion Energy thermal break concept monoblock.

In this work we propose the recently introduced Neural Network Filtered Back-Projection (NN-FBP) method^42^ to reduce the acquisition time in NT experiments. At the best of our knowledge, this is the first study which proposes and tests a ML based reconstruction method for NT. The NN-FBP method avoids to a degree the aforementioned problems of analytical and iterative reconstruction algorithms. In fact, NN-FBP is faster than iterative methods, since it has similar computation complexity to FBP, and learns how to use problem specific knowledge, providing high image quality even for limited datasets. We demonstrate that this method is suitable for neutron data and outperforms conventional reconstruction methods used in NT. Furthermore, the NN-FBP method can reliably reduce the scan time, reconstruction time and the amount of data storage. As case study, we chose to inspect part of a monoblock (Fig. 1) from the divertor region of a fusion energy device by means of Sparse-View NT and the NN-FBP reconstruction algorithm. The main motivation of employing the fusion divertor monoblock as a specimen is because of the large number of armor that will be required for the divertor assembly within the ITER project^43^ and consequently matches the need of a quality check technique. The structural integrity of these samples subjected to high thermal loads is fundamental within a tokamak fusion energy device. A comparative study between X-ray CT and NT has been recently carried out^44^ to inspect the quality of manufactured monoblocks.

In our work, simulated and real neutron data were used to assess the performances of the NN-FBP, FBP and Simultaneous Iterative Reconstruction Technique (SIRT)^45^ methods as a function of the number of projections. The reconstructed images were quantitatively compared in terms of several image quality indexes.

## Results

The NN-FBP method combines different FBP reconstructions, each with a custom filter, to produce a single image. The filters are determined by training an Artificial Neural Network (ANN). A brief description of the algorithm and of the ANN architecture is reported in the Methods section. The network input is a vector that contains the projection data and the network output is a single reconstructed pixel. The intermediate hidden layer of the network consists of *N*_*h*_ hidden nodes. This parameter can be chosen freely and, in the NN-FBP implementation, represents the number of different FBP reconstructions to compute and combine in a single image. We used simulated data to find the optimal value of *N*_*h*_ which ensures the best balance between reconstructed image quality and reconstruction time. We underline that the network must be re-trained to change the number of hidden nodes.

**Figure 2:**
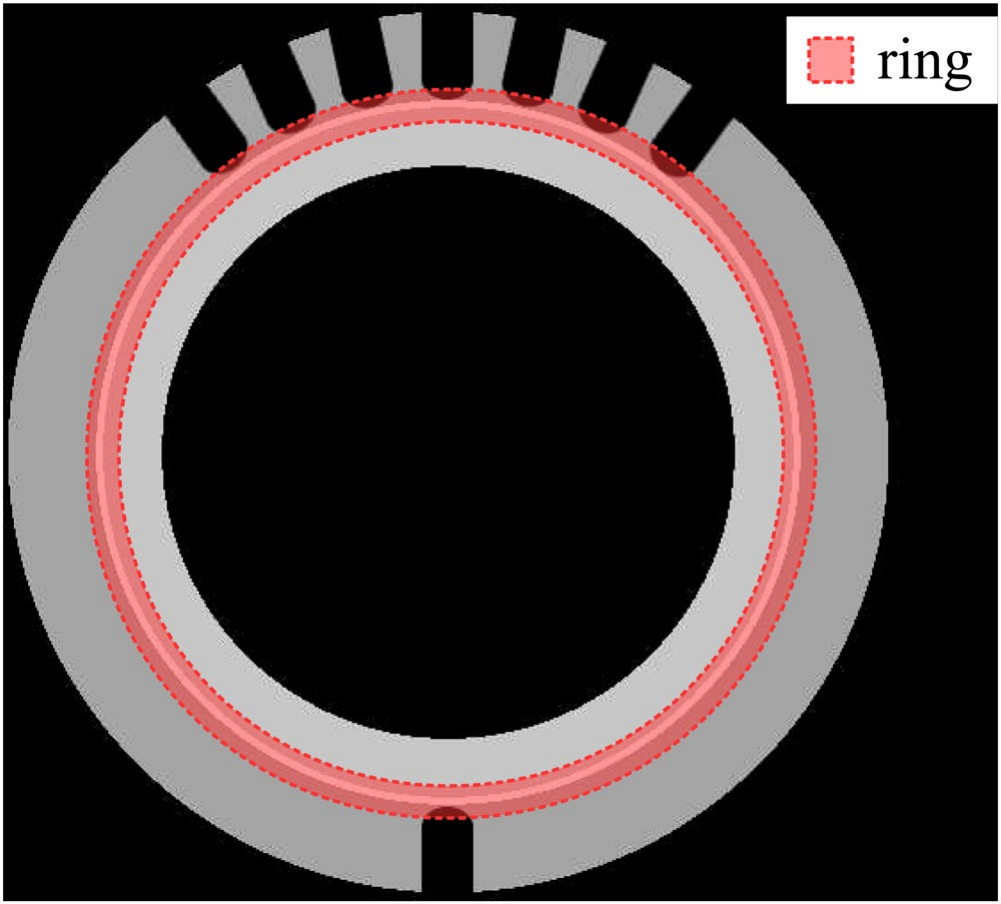
Diagram showing a slice of the simulated phantom and the ring-shaped ROI (red area) used for the computation of the NRMSE.

Afterwards, we quantitatively compared the NN-FBP, FBP and SIRT methods as a function of the number of projections using both simulated and real data. The evaluation of the image quality was carried out by computing the Normalized Root Mean Square Error (NRMSE), the Structural Similarity Index (SSIM)^46^, the Feature Similarity Index (FSIM)^47^ and the Gradient Magnitude Similarity Deviation (GMSD)^48^. The NRMSE is a measure of the reconstruction error and it is defined as:

1 $${\rm{NRMSE}}=\frac{||{I}_{{\rm{rec}}}-{I}_{{\rm{gt}}}{||}_{2}}{||{I}_{{\rm{gt}}}{||}_{2}}$$

where *I*_rec_ and *I*_gt_ are vectors containing pixel values of the reconstructed and ground truth image, respectively, $||\cdot {||}_{2}$ is the Euclidean norm. In our analysis, the NRMSE was computed both on the sample (NRMSE sample) and on a ring-shaped region of interest (NRMSE ring) shown in Fig. 2 in order to evaluate the reconstruction accuracy of a particular thin feature of the specimen. The sample mask was computed using the Otsu’s thresholding method^49^. The SSIM index quantifies the structural similarity between two images by comparing the luminance, the contrast and the structure. The SSIM value ranges from −1 to 1, a higher value indicates superior image quality. The FSIM is an image quality index that better reflects the perception of the human visual system evaluating salient low-level image features. In fact, FSIM index exploits the phase congruency and the image gradient magnitude, which are complementary features in characterizing the image quality. The FSIM value ranges from 0 to 1, a higher value indicates superior image quality. The GMSD index measures the variation in the similarity of gradient maps between two images. We used this metric to assess the quality of the edges. GMSD values lie between 0 and 1, a value closer to 0 indicates better similarity in the gradient maps.

### Simulation study

A numerical phantom, which mimics the Cu-CuCrZr pipe (Fig. 1), was generated to find optimal parameters for the reconstruction and to test the NN-FBP method. A slice of the numerical phantom is shown in Fig. 2. Simulated projections were obtained by computing the Radon Transform of the phantom image, assuming a parallel beam geometry. First, we reconstruct images from an over-sampled dataset of 1335 projections using the SIRT method with 400 iterations. The over-sampled dataset contains twice the number of projections required by the Nyquist-Shannon condition. In fact, the sampling theorem is exactly satisfied for 668 projections (the widest horizontal dimension of the sample is ~430 pixels long). We then train the ANN to mimic the reconstructed images obtained from the oversampled dataset, using a subset of the available projections. The network was trained on 10^5^ pixels/slice from 10 training images and 10^5^ pixels/slice from 10 validation images. The image quality indexes were evaluated on 30 reconstructed images of a numerical phantom that differs from spatial orientation from the one used for training. We used the original phantom images as ground truth image.

**Figure 3:**
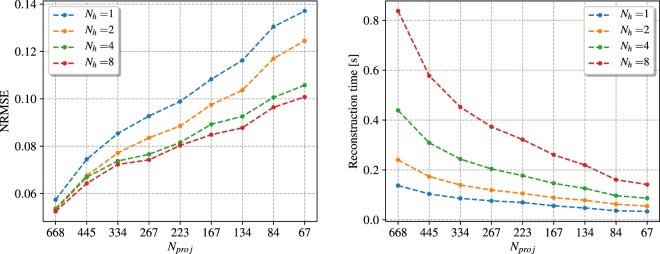
(left) The NRMSE values and (right) the reconstruction time for the number of hidden nodes 1, 2, 4 and 8, as a function of the number of projections.

Firstly, we evaluated the quality of the NN-FBP reconstruction for different number of hidden nodes (*N*_*h*_). Figure 3 shows the NRMSE computed over the whole image and the reconstruction time as a function of the number of projections (*N*_*proj*_). Each line represents reconstructions with 1, 2, 4 and 8 hidden nodes. It is clear that in general higher reconstructed image quality is achieved by increasing the number of hidden nodes, but at the expense of a longer reconstruction time. Hence, we chose to use 4 hidden nodes in both simulated and experimental study, since it ensures a good balance between image quality and short reconstruction time (less than 300 ms for under-sampled datasets).

**Figure 4:**
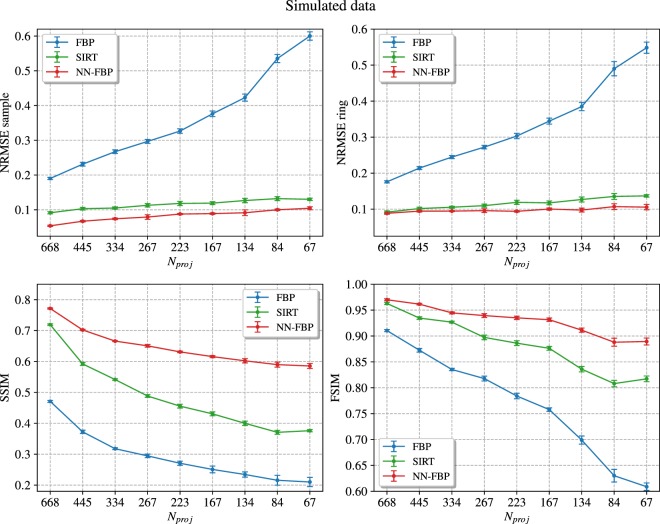
Comparison of different image quality indexes computed from FBP, SIRT and NN-FBP reconstructions of simulated data, as a function of the number of projections *N*_*proj*_. (top-left) The NRMSE evaluated over the sample mask, (top-right) the NRMSE evaluated within the ring-shaped ROI, (bottom-left) the SSIM index and (bottom-right) the FSIM index. The error bars indicate three standard deviations.

Afterwards, we compared the reconstruction quality of the NN-FBP with respect to the quality of conventional algorithms SIRT and FBP, in terms of the aforementioned image quality indexes. In our analysis, all FBP reconstructions were performed with the Ram-Lak filter. Figure 4 shows the NRMSE sample (top-left), the NRMSE ring (top-right), the SSIM (bottom-left) and the FSIM (bottom-right) evaluated from FBP, SIRT and NN-FBP reconstructions of simulated data as a function of the number of projections. It is clear that NN-FBP method outperforms significantly the FBP and SIRT. In fact, the indexes related to NN-FBP reconstructions indicate better image quality than conventional algorithms for all number of projections considered. The FSIM turns out to be the most significant image quality index. It is evident from the FSIM plot in Fig. 4 that the number of projections can be reduced using the NN-FBP method to 134, i.e. 1/8 of the over-sampled dataset and 1/4 of the projections required by the sampling theorem, ensuring image quality comparable to FBP reconstruction for *N*_*proj*_ = 668.

### Experimental study

Neutron images of the sample were acquired at the IMAT beamline^50–52^, ISIS neutron spallation source, Rutherford Appleton Laboratory, U.K. We performed tomographic scans of two similar samples by collecting an over-sampled dataset of 1335 projections in the angular range [0°, 360°) with 30 s exposure time per projection, which is the maximum exposure time per projection allowed by the used camera. A complete CT scan takes approximately 11 hours. Our setup, described in detail in the section Methods, provides a number of neutrons per pixel equals to 1.5 ⋅ 10^3^.

Also in this case, the oversampled datasets contains twice the number of projections required by the sampling theorem. The first sample was used to train the ANNs, the latter to evaluate the image quality of the NN-FBP reconstructions. The network was trained to mimic images obtained from 1335 projections using the SIRT method with 400 iteration. In particular, 10^5^ pixels/slice from 10 training images and 10^5^ pixels/slice from 10 validation images of the first sample were used to train the ANNs. We evaluated the image quality indexes on 30 reconstructed images of the second sample for each reconstruction method. At this stage, we regard as ground truth images the SIRT reconstruction of the oversampled dataset (*N*_*proj*_ = 1335) with 400 iterations.

**Figure 5:**
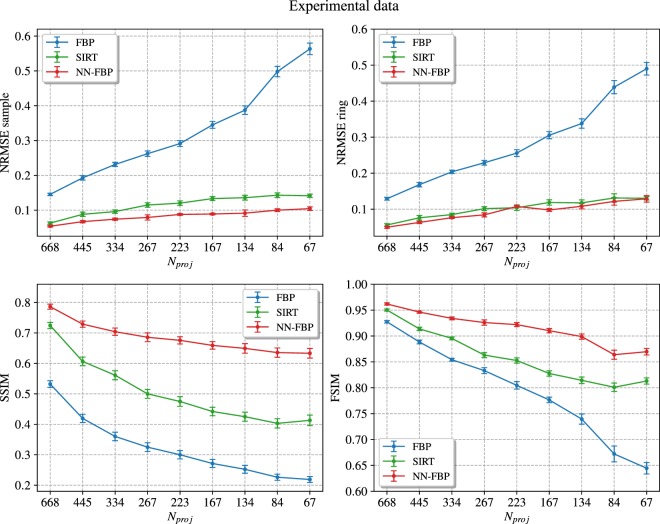
Comparison of different image quality indexes computed from FBP, SIRT and NN-FBP reconstructions of real data, as a function of the number of projections *N*_*proj*_. (top-left) The NRMSE evaluated over the sample mask, (top-right) the NRMSE evaluated within the ring-shaped ROI, (bottom-left) the SSIM index and (bottom-right) the FSIM index. The error bars indicate three standard deviations.

Figure 5 shows the NRMSE sample (top-left), the NRMSE ring (top-right), the SSIM (bottom-left) and the FSIM (bottom-right) evaluated from FBP, SIRT and NN-FBP reconstructions of experimental data as a function of the number of projections. In general, the trend of each index obtained in the experimental study is quite similar to the results of the simulation study. In fact, the NN-FBP shows higher image quality than FBP and SIRT in terms of the NRMSE sample, SSIM and FSIM for all numbers of projections. From the NRMSE ring plot, we deduce that NN-FBP method provides at worst reconstruction comparable for accuracy to the SIRT. From the FSIM plot in Fig. 5 we conclude that the number of projections can be reduced using the NN-FBP method to 223, i.e. 1/6 of the over-sampled dataset and 1/3 of the projections required by the sampling theorem, ensuring image quality comparable to the standard FBP reconstruction for *N*_*proj*_ = 668.

**Figure 6:**
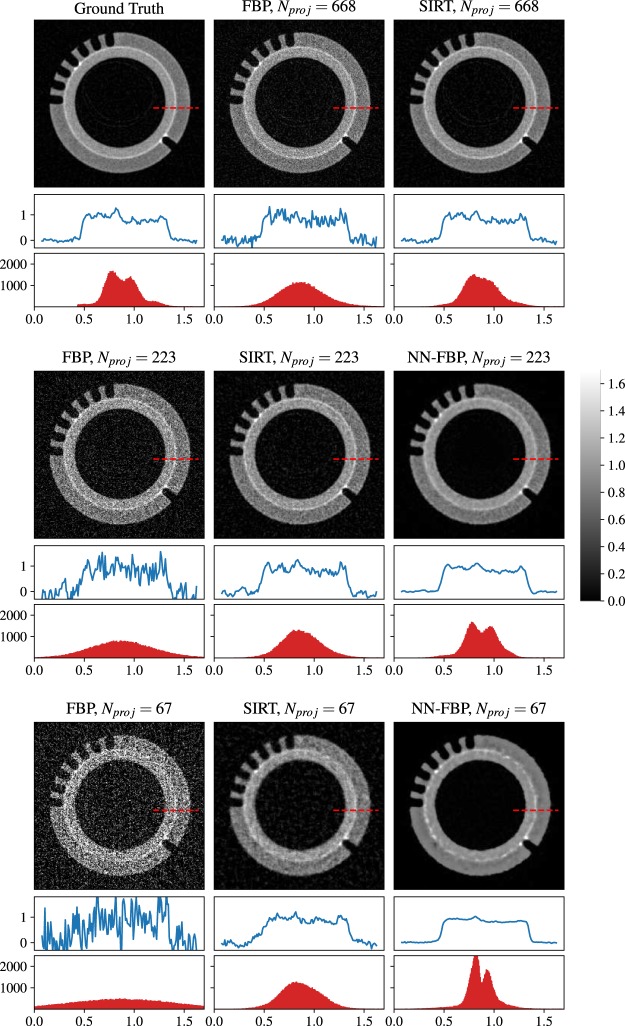
A comparison of CT reconstructed images of real data obtained using FBP, SIRT and NN-FBP methods for different number of projections. In the reading order: the ground truth image obtained with SIRT algorithm (*N*_*proj*_ = 1335 and 400 iterations), the FBP reconstruction for *N*_*proj*_ = 668 (matching exactly the Nyquist condition), the SIRT reconstruction for *N*_*proj*_ = 668 and 400 iterations, the FBP reconstruction for *N*_*proj*_ = 223, the SIRT reconstruction for *N*_*proj*_ = 223 and 400 iterations, the NN-FBP reconstruction for *N*_*proj*_ = 223, the FBP reconstruction for *N*_*proj*_ = 67, the SIRT reconstruction for *N*_*proj*_ = 67 and 400 iterations, the NN-FBP reconstruction for *N*_*proj*_ = 67. Below each image is shown the intensity profile along a line segment marked in each CT slice with a red dashed line. The intensity values are represented in the range [−0.3, 1.8] cm^−1^ and the segment length is 160 pixels. Below each intensity profile the histogram of the attenuation coefficient values within the sample is represented in the range [0, 1.7] cm^−1^.

In Fig. 6 we show a comparison of different reconstructed slices: the ground truth image, the FBP and SIRT reconstructions of 668 projections, matching exactly the Nyquist condition, and the FBP, SIRT and NN-FBP reconstructions for 223 and 67 projections. Below each image is shown the intensity profile along a line segment marked in each CT slice with a red dashed line. Furthermore the histogram of attenuation coefficients within the sample mask is represented below each intensity profile plot. We note from a visual inspection that for 223 projections the FBP reconstruction is affected by high noise dose which makes the segmentation not feasible. On the other hand, the NN-FBP method with 223 projections provides high contrast images and less noise than conventional algorithms. Furthermore, we note that the NN-FBP for *N*_*proj*_ = 223 is the only one method able to reconstruct images with a multimodal distribution of the pixel values. The edges and the sample features are accurately reconstructed. This result indicates that segmentation and analysis can be actually performed on a NN-FBP reconstruction of a limited dataset with 223 projections. When the number of projection is reduced to 67 (1/10 of the required one by the sampling theorem) the NN-FBP reconstructs well the strong edges but with an over-smoothing which suppresses low contrast structure. Hence the severe under-sampling in NN-FBP method leads to low-noise images but with a loose of image features.

**Figure 7:**
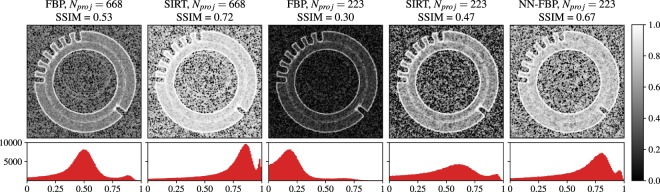
The SSIM maps computed from FBP, SIRT and NN-FBP reconstructions of real data for *N*_*proj*_ = 668 and *N*_*proj*_ = 223 with respect to the ground truth image. Below each image the histogram of local SSIM values is represented, while above the global SSIM value is reported.

To assess the local structural similarity of the reconstructed images with respect to the ground truth image we computed the local SSIM map. In Fig. 7 we show the SSIM maps related to the FBP and SIRT reconstructions of 668 projections, the FBP, SIRT and NN-FBP reconstructions of 223 projections. The histogram of local SSIM values is represented below each image and the global SSIM is also reported. We observe that in the case of the NN-FBP method with 223 projections the majority of local SSIM values range from 0.7 to 1 and globally around 0.67. This result is significantly better than the results obtained from FBP and SIRT for the same number of projections (i.e. global SSIM 0.30 and 0.47 for FBP and SIRT and majority of local SSIM values <0.7). Furthermore, the NN-FBP reconstruction of 223 projections outperforms the standard FBP reconstruction of 668 projections in terms of local and global SSIM values. However, the SIRT reconstruction of 668 projections shows slightly better structural similarity respect the ground image than the NN-FBP reconstruction for 223 projections. In fact, the global SSIM for the SIRT image is 0.72 while for NN-FBP image is 0.67.

**Figure 8:**
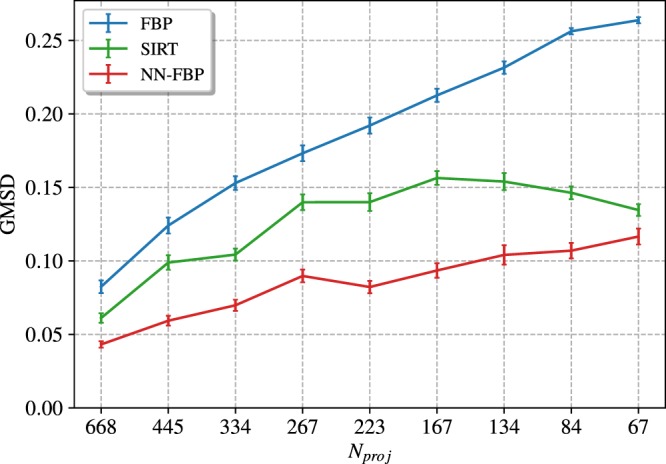
Comparison of the GMSD values computed with respect to the ground truth image from FBP, SIRT and NN-FBP reconstructions of real data as a function of the number of projections. The error bars indicate three standard deviations.

In Fig. 8 we show a comparison of the GMSD values computed with respect to the ground truth image for each reconstruction algorithm as a function of the number of projections. We observe that for each reconstruction method the edge quality decreases when the number of projections is reduced. However, the NN-FBP outperforms SIRT and FBP in terms of the GMSD values for each number of projections considered. Furthermore, the edge quality of the NN-FBP reconstruction of 223 projections is comparable to the standard FBP reconstruction of 668 projections.

**Figure 9:**
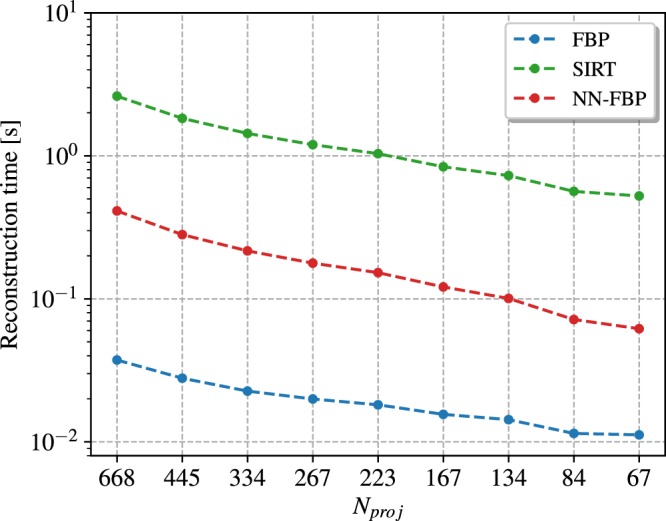
The average reconstruction time per slice of the FBP, SIRT and NN-FBP method as a function of the number of projections. All reconstructions were performed on GPU.

Finally, we evaluated the average reconstruction time per slice of the FBP, SIRT and NN-FBP methods as a function of the number of projections. The results are shown in Fig. 9. The FBP method is the fastest reconstruction algorithm. However, NN-FBP is in general one order of magnitude faster than SIRT and one order of magnitude slower than FBP, ensuring reconstruction time per slice of the order of tenths of a second. In Table 1 we report the training time of the NN-FBP method as a function of the number of projections and for different number of hidden nodes. Obviously the training time increases with the amount of training and validation data and in our analysis we fixed them. For each training stage we used 10^5^ pixels/slice from 10 training images and 10^5^ pixels/slice from 10 validation images. We observe from Table 1 that the training time increases with the *N*_*h*_ value but does not strictly depend on the number of projections. In general, the training task requires tens of minutes which is a reasonable time with respect to the acquisition time of a NT scan.

**Table 1:** Training time of the NN-FBP method as a function of the number of projections and for different number of hidden nodes.

*N*_*proj*_	*N*_*h*_ = 1	*N*_*h*_ = 4	*Nh* = 16
668	155 s	358 s	898 s
445	150 s	260 s	555 s
334	71 s	187 s	594 s
267	136 s	451 s	656 s
223	97 s	307 s	551 s
167	95 s	384 s	420 s
134	90 s	321 s	1209 s
84	100 s	317 s	915 s
67	94 s	402 s	857 s

## Discussion and Conclusions

We have studied for the first time the performance of the NN-FBP method with neutron data and compared to conventional reconstruction algorithms used in NT in terms of different image quality metrics. We demonstrate that NN-FBP method outperforms the FBP and SIRT ones with respect to image quality. Furthermore, the computation complexity of NN-FBP method is lower than SIRT. Hence, NN-FBP method provides reconstructions in shorter times with respect to iterative methods. We conclude that the NN-FBP can reliably reduce scan time, reconstruction time and data storage providing high image quality for sparse-view NT. Specific prior knowledge is not explicitly moulded in the NN-FBP method, as opposed to advanced iterative reconstruction algorithms. In fact the method learns the features of the training images by tuning the neural network’s weights appropriately. Hence the NN-FBP method can be implemented with high computational efficiency at neutron imaging facilities for the broader applicability than regularized iterative reconstruction algorithms.

The main requirement of the NN-FBP method is that the scanned objects should consist only of the same materials present in the training samples. When this prerequisite is satisfied the NN-FBP method is able to reconstruct accurately objects with different shape and size of the training samples^53^.

Our experimental study demonstrates that using the NN-FBP method, the number of projections can be reduced to 1/3 of the projections required by the sampling theorem, ensuring image quality comparable to standard FBP reconstruction. Hence, the acquisition time can be reduced to 1/3 of the time requested by a standard CT scan. However, the reconstruction quality of the NN-FBP is highly dependent on the quality of the projections and reconstructed images used in the training stage. In principle, better results can be obtained by optimizing the imaging setup to increase the signal-to-noise ratio of neutron projections.

In this study, we focused on the application of the NN-FBP method to sparse-view CT reconstruction of objects similar to a training sample, which was scanned over a large number of view angles. The NN-FBP was trained using the SIRT reconstruction of the over-sampled training dataset. However, several experimental situations limit the angles for which projection data can be acquired. The NN-FBP method can be used in these cases to emulate an advanced but slow regularized iterative method to produce reconstructions from limited projection data. In particular, this can be of great interest for spatio-temporal reconstruction of dynamic systems. For example, the NN-FBP method could be used to study the dynamics of slow periodic phenomena in a stroboscopic mode by acquiring projections according to a Golden ratio based sequence^54^. The training should be performed on high-quality reconstruction of the system at particular time instant. The temporal evolution can be reconstructed with NN-FBP if the aforementioned prerequisite is satisfied during the experiment. However the feasibility of these applications in NT remains subject of further research.

The NN-FBP can be used to reconstruct also *truncated data*, occurring when the scanned object is larger than the field-of-view (FOV) of the imaging system. Truncated sinograms can lead to strong artifacts in the reconstructed images. When using the FBP method with truncated data, the artifacts can be reduced by replicating the projection boundary values to form a larger virtual detector^55^. This method cannot be applied to iterative algorithms, which require projections of the entire sample. Conversely, the padding approach can be used with the NN-FBP method since it is based on FBP reconstructions with custom filters.

We think that the NN-FBP could be improved by using deeper networks with the aim of learning more features of the sample. Deep learning and machine learning in general are promising and innovative approaches for image reconstruction. This field of research nowadays is of interest in medical and X-ray imaging^56^, but we think that also the NI community should take into account new ML based reconstruction theories and techniques.

## Methods

### Sample

Fabrication of the Cu-CuCrZr pipe, shown in Fig. 1, was carried out in the following way. Firstly, the inner CuCrZr pipe with a thickness of 1 mm was wrapped in three turns of a 25 *μ*m thick braze foil to a total thickness of 75 *μ*m. The braze foil is a 50:50 copper-gold mix known commercially as Orobraze^TM^. Next, two half copper pipe ‘sleeves’ were placed around the inner pipe. The sleeves were held in place by tying them with a molybdenum wire in several locations along the length of the pipe. This assembly was heated in a vacuum furnace to perform the brazing cycle and join the inner and outer pipes. Finally, the molybdenum wire was removed, and 1 mm wide grooves were machined along the length of the copper pipe; one groove along one side and seven equidistant grooves on the opposing side. For the purpose of this investigation, a length of 20 mm pipe was cut from a longer part.

### Overview of the approach

The NN-FBP method is based on a nonlinear weighted sum of different FBP reconstructions, each of these with a specific filter. A neural network model is exploited to train these custom filters. The type of network used for the NN-FBP is the multilayer perceptron^57^. This network has three layers: the input layer, the hidden layer and the output layer, each of them composed of *n*, *N*_*h*_ and *m* nodes, respectively. In a multilayer perceptron, each input node is connected to all hidden nodes with a weight *w*_*ij*_, and each hidden node to all output nodes with a weight *q*_*ij*_. Hence, the connections between the input layer and the hidden layer is described by the *n* × *N*_*h*_ matrix **W**, containing the *w*_*ij*_ weights. The *m* × *N*_*h*_ matrix **Q** containing the weights *q*_*ij*_ represents the connections between the hidden nodes and the output nodes. Scalar values are subtracted from the output of each hidden and output node. Moreover, a logistic function *σ* is applied as activation function to the output of each hidden and output node, making the neural network a nonlinear model. The number of hidden nodes *N*_*h*_ is a free parameter, to be determined for each specific problem. The output vector **O** of a multilayer perceptron, with *N*_*h*_ number of hidden nodes, for the input vector **z** can be expressed as:

2 $${{\bf{O}}}_{{\bf{Q}},{\bf{W}},{\bf{b}},{{\bf{b}}}_{0}}({\bf{z}})=\sigma (\sum _{i=1}^{{N}_{h}}{{\bf{q}}}_{i}\sigma ({{\bf{w}}}_{i}\cdot {\bf{z}}-{{\bf{b}}}_{i})-{{\bf{b}}}_{0})$$

where **w**_**i**_ and **q**_**i**_ are single columns of the matrices **W** and **Q** respectively, while **b**_**i**_ and **b**_**0**_ are the bias weights. According to supervised learning approach, an unknown function can be approximated by an ANN if the output values **f**_**i**_ are known for a particular set of *T* input vectors **z**_**i**_. Hence, the network weights are found in a training task that consists in minimize the cost function:

3 $$e({\bf{Q}},{\bf{W}},{\bf{b}},{{\bf{b}}}_{0})=\sum _{i=1}^{T}{({\bf{O}}({{\bf{z}}}_{i})-{{\bf{f}}}_{i})}^{2}.$$

In the case of the NN-FBP, the input vector has the same size of the detector array, composed of *N*_*d*_ elements each with coordinate *τ*_*d*_. The input vector components can be expressed as follows:

4 $$z({\tau }_{d})=\sum _{k}{P}_{{\theta }_{k}}({x}_{i}\,\cos \,{\theta }_{k}+{y}_{i}\,\sin \,{\theta }_{k}-{\tau }_{d})$$

while output layer is composed of a single node and described by the formula:

5 $${O}_{{\bf{Q}},{\bf{W}},{\bf{b}},{b}_{0}}({\bf{z}})=\sigma (\sum _{j=1}^{{N}_{h}}{q}_{j}\sigma (FB{P}_{{{\bf{w}}}_{j}}({x}_{i},{y}_{i})-{{\bf{b}}}_{j})-{b}_{0}).$$

The output of the neural network can be viewed as weighted sum of *N*_*h*_ FBP reconstructions with custom filters and specific biases. Hence, the computational complexity of the NN-FBP method depends on the number of hidden nodes *N*_*h*_, but is comparable to the FBP method.

### Tomographic acquisition at the IMAT beamline

The data acquisition was carried out at the IMAT beamline, ISIS neutron spallation source, Rutherford Appleton Laboratory, U.K. The sample was placed on the rotating platform at the distance *L* =  10 m from the beam aperture and at the distance *d* =  25 mm from the scintillator screen. The diameter of the beam aperture was *D* =  40 mm, resulting in a L/D ratio of 250. The neutron flux for this setup is 5.9 ⋅ 10^6^ n/cm^2^/s^51^. The imaging system consisted of a CMOS camera with 2048 × 2048 pixels coupled with optical lenses and a scintillator ^6^LiF/ZnS with thickness of 50 *μ*m. The FOV was set to 59.5 × 59.5 mm^2^ and the resulting pixel size was 29 *μ*m. Each tomographic scan was performed by collecting a set of 1335 radiographs in the angular range [0°, 360°), with an exposure time of 30 s for each projection and an overall scan time of approximately 11 hours. Open beam and dark field images were taken as well in order to perform the data normalization.

### Data processing and reconstruction

The acquired raw projections were normalized respect to the dark images, flat field images and to the neutron dose. Afterwards, the normalized projections were pre-processed by removing dead-pixels and gamma-spots, while ring artifacts were suppressed by means of a filter based on combined wavelet and Fourier analysis^58^.

In the simulation experiment, we generated images of 3480 × 3480 pixels representing a slice of the sample (Fig. 2). We evaluated equispaced projections in the angular range [0°, 360°) for a detector with 3480 pixels. We assumed a parallel beam geometry which is a fair approximation for neutron beams characterized by an L/D ratio of 250. Afterwards, we rebinned the projected data to 870 pixels and we added Poisson noise assuming 5000 counts as background intensity. The reconstruction was done on a 870 × 870 pixels grid.

Pre-processing, reconstruction and analysis of simulated and real data were performed using a Python code developed *ad hoc*. For the CT reconstruction task we exploit the ASTRA toolbox^59,60^. All reconstructions and simulations were performed on a Linux workstation equipped with an Intel Core i7-6700HQ CPU @ 3.40 GHz CPU, 64 GB of system RAM and a NVIDIA GTX TITAN X GPU.

## Data Availability

The datasets generated during and/or analysed during the current study are available from the corresponding author on reasonable request.
